# Oral Dydrogesterone Versus Vaginal Progesterone for Luteal Phase Support in Frozen–Thawed Embryo Transfer Cycles: A Systematic Review and Meta-Analysis of Randomized Controlled Trials

**DOI:** 10.3390/jcm14093238

**Published:** 2025-05-07

**Authors:** Konstantinos Stavridis, Dimitrios Balafoutas, Theodoros Kalampokas, Vassiliki Benetou, Evangelia Samoli, Nikolaos Vlahos, Maria-Iosifina Kasdagli

**Affiliations:** 1 2nd Department of Obstetrics and Gynecology, “Aretaieion” University Hospital, 11528 Athens, Greece; dbalafoutas@yahoo.com (D.B.); kalamp@yahoo.com (T.K.); nikosvlahos@med.uoa.gr (N.V.); 2 Department of Hygiene, Epidemiology and Medical Statistics, Medical School, National and Kapodistrian University of Athens, 11527 Athens, Greece; vbenetou@med.uoa.gr (V.B.); esamoli@med.uoa.gr (E.S.); kasdaglimar@med.uoa.gr (M.-I.K.)

**Keywords:** oral dydrogesterone, frozen embryo transfer, luteal support, vaginal progesterone, meta-analysis

## Abstract

**Background/Objectives**: Until recently, oral dydrogesterone has only been established in fresh in vitro fertilization (IVF) cycles, whereas its role in luteal phase support (LPS) for frozen embryo transfer (FET) cycles remains unclear. The aim of this study is to determine whether oral dydrogesterone as LPS in FET cycles results in pregnancy rates comparable to vaginal progesterone, focusing primarily on ongoing pregnancy rates, but also on clinical pregnancy, miscarriage, and live birth rates. **Methods**: The study followed the Preferred Reporting Items for Systematic Reviews and Meta-Analyses guidelines. Five databases (Embase, MEDLINE^®^, APA PsycInfo, Global Health, and HMIC) and two additional sources were searched from inception to November 28, 2024. Only randomized controlled trials (RCTs) were included. A common effects model combined risk estimates, and heterogeneity was assessed using I^2^. Study quality was evaluated with Risk of Bias 2 (RoB2), and evidence certainty was graded using GRADE. **Results**: Overall, five RCTs with a total of 636 women were included in the meta-analysis. The comparison between oral dydrogesterone and vaginal progesterone for LPS did not yield significant differences for any of the outcomes studied. For ongoing pregnancies, the pooled odds ratio (OR) was 0.90 (95% CI: 0.59–1.35), with no heterogeneity (I^2^ = 8.7%). For miscarriage events, the OR was 1.41 (95% CI: 0.63–3.13, I^2^ = 0). For clinical pregnancies, the OR was 0.94 (95% CI: 0.62–1.42, I^2^ = 49.2%), with heterogeneity attributed to dosage. For live births, the pooled OR was 1.08 (95% CI: 0.67–1.75, I^2^ = 0%). Two studies were assessed as high risk of bias, two as low risk, and one as moderate. The GRADE assessment indicated low to moderate certainty of evidence. **Conclusions**: Oral dydrogesterone and vaginal progesterone yield comparable reproductive outcomes for LPS in FET cycles. Given its ease of administration, dydrogesterone may serve as a viable alternative in future FET protocols. However, further RCTs are needed to assess its efficacy against other progesterone administration routes.

## 1. Introduction

Over the last decades, frozen embryo transfer (FET) has been increasingly adopted in modern fertility units. The Human Fertilisation and Embryology Authority highlighted that between 2013 and 2018, fresh embryo transfers decreased by 11%, while FET cycles nearly doubled and accounted for almost 40% of all in vitro fertilization (IVF) cycles in 2018 [1]. In the USA, Australia, and New Zealand, the number of thawed transfers started to prevail over fresh cycles [2]. The European fertility units, although clearly delayed, followed this trend by increasing the rate of FET cycles from 28% to 34% between 2010 and 2016 [3]. Several factors contribute to the extreme rise of FET cycles over the years. First, a more efficient and safe warming and vitrification technique has been established. Furthermore, it has been proposed that FET cycles are linked to a lower likelihood of ovarian hyperstimulation syndrome (OHSS) associated with controlled ovarian stimulation (COS) [4,5,6]. Lastly, the routine use of pre-implantation genetic testing for aneuploidies (PGT-A) and the subsequent better genetic counseling for fertility couples contributed further to the extreme rise of FET cycles. In these cases, the embryos could remain frozen until chromosomal abnormalities have been excluded [2].

In FET cycles, progesterone can be administered via five different routes, including vaginal (PV), oral, intramuscular (IM), rectal, and subcutaneous. To date, no specific guidelines have been established to indicate the superiority of any of the aforementioned routes of progesterone administration in terms of reproductive rates. Nevertheless, various studies have compared other treatment routes with the most popular one, which is the vaginal route. In a three-arm randomized control trial (RCT) published in 2021, Devine and colleagues assessed the use of PV alone (200 mg twice daily), the combination treatment of PV (200 mg twice daily) with IM injections every 3rd day (50 mg), and the use of IM injections alone (50 mg daily), in terms of live birth rates among 1125 women planning vitrified-warmed transfer of high-quality non-biopsied blastocysts. The authors concluded that the PV-only treatment was associated with poor pregnancy rates, which was reflected by the high miscarriage rates, leading to the exclusion of the PV-only treatment group from the rest of the study. However, higher doses of 400 mg of progesterone daily, as performed in many European studies, might have yielded improved reproductive outcomes [7].

When comparing the most widely used methods of administration, it is essential to quantify and consider their adverse effects. Vaginal irritation, discharge, and bleeding are the most common side effects described for the vaginal administration, whilst intramuscular injections are associated with local pain, discomfort, and skin inflammation [8,9]. In this context, the oral route of progesterone administration seems an option for luteal phase support, as it is considered more patient-friendly and easy to use [10]. In fresh cycles, prospective studies have indicated that oral dydrogesterone is as effective as vaginal treatment with comparable patient satisfaction and tolerability [11,12,13]. In FET cycles, however, the role of oral dydrogesterone compared to the gold-standard, the vaginal route, remains inconclusive.

Based on the above, the aim of this current systematic review and meta-analysis is to compare oral dydrogesterone with vaginal progesterone in FET cycles regarding pregnancy outcome rates.

## 2. Materials and Methods

The protocol is published in the International Prospective Register of Systematic Reviews (PROSPERO) with reference number (CRD42023489318), while no deviations from the original protocol will be presented in the current manuscript.

### 2.1. Information Sources (Search Strategy)

The systematic review was conducted according to the guidelines of the Preferred Reporting Items of Systematic Reviews and Meta-analyses (PRISMA) [14] (PRISMA Checklist, Supplementary Material). We performed a literature search for relevant studies from inception to 28 November 2024 across five databases: Embase (OVID), MEDLINE (OVID), APA PsycInfo (OVID), Global Health (OVID), and Health Management Information Consortium (HMIC) (OVID). Additional records were searched through registers, including Research Square and MedRxiv. The MedRxiv search was simplified according to database search functionality.

The following search terms were combined in Ovid: “dydrogesterone”, “DYD”, “micronized progesterone”, “MVP”, “luteal phase support”, “ART”, “Assisted reproduction”, “IVF”, “In Vitro fertilization”, “Frozen thawed”, “FET”. Cross references were hand-searched to ensure that no study had been missed. We (K.S and M.I.K) independently reviewed the titles and abstracts and further assessed the full-text of the articles for compliance with the inclusion and exclusion criteria. Any disagreement between the authors was resolved by discussion.

### 2.2. Inclusion and Exclusion Criteria

We applied the following eligibility criteria structured by PICOS items:

Population: females undergoing frozen embryo transfer cycles;

Intervention: oral dydrogesterone;

Comparator: vaginal progesterone;

Outcome: The reported primary outcome of interest was the ongoing pregnancy rate (OPR), defined as a viable pregnancy beyond 12 weeks of gestational age. The secondary outcomes considered were clinical pregnancy rate (CPR), miscarriage rate (MR), and live birth rate (LBR). Clinical pregnancy was evaluated by ultrasonographic visualization of ≥1 gestational sac or clinical signs of pregnancy (visualization of fetal heartbeat). Miscarriage was defined as a spontaneous loss of an intra-uterine pregnancy before week 12 of pregnancy.

### 2.3. Study Design

RCTs with women undergoing frozen embryo transfer cycles. Reviews, meta-analyses, cohort studies, case-control studies, case series, case reports, commentaries, editorials, and conference abstracts, and non-English language manuscripts were excluded.

### 2.4. Data Extraction

Data from selected publications were extracted on first author’s name, publication year, country, sample size, numbers lost to follow-up (post withdrawals), type of FET (e.g., programmed/artificial cycle or natural/mNC), numbers of patients in the two comparator groups (oral and vaginal progesterone), maternal age (in years as mean ± standard deviation (SD)) per group, Body Mass Index (BMI) (in kg/m^2^ as mean ± SD) per group, progesterone dosage per group, and outcome data as number of cases per treatment group (Table 1).

### 2.5. Statistical Analysis

As the outcome variables were dichotomous, namely positive pregnancy (either clinical, ongoing, live birth, or a miscarriage) or not, the odds ratio (OR), calculated from tables after raw data were extracted from each paper, was used as an effect measure with associated standard error. We applied a common effect model with inverse variance and assessed the heterogeneity across studies using the I^2^ metric [15] In cases where an I^2^ over 50% was estimated, indicating substantial heterogeneity between the study effects estimates, a random effects model was applied; otherwise, a common effects model was used.

We investigated heterogeneity by sub-group analysis based on dydrogesterone clinical dosage. To evaluate the potential influence of the study by Ozer et al. [16], which utilized an mNC-FET, we performed a leave-one-out meta-analysis for each outcome reported by this study. All statistical analyses were performed using STATA software v13.

### 2.6. Risk of Bias (ROB) and Certainty of Evidence

The risk of bias was assessed by two independent reviewers (K.S and M.I.K), using the Risk of Bias 2 (RoB 2) tool for randomized studies [15]. A study could be of “high risk of bias”, “some concerns”, or “low risk of bias” [15]. Risk-of-bias assessment figures were created via the web-app RobVis (Risk-Of-Bias VISualization) [17].

We assessed the certainty of evidence using the GRADE framework, categorized as high, moderate, low, or very low [18].

**Table 1:** Characteristics of the studies included in the meta-analysis.

Author, Year	Location	Time Period	Sample Size (N, Total)	Type of FET	N, Oral P4	N, Vaginal P4	Age (Mean ± SD), Oral P4	Age (Mean ± SD), Vaginal P4	BMI (Mean ± SD), Oral P4	BMI (Mean ± SD), Vaginal P4	Oral P4 Daily Dosage	Vaginal P4 Daily Dosage
Macedo et al. [19], 2023	Brazil	2019–2021	73	artificial	36	37	33.2 ± 4.4	34.1 ± 4.4	25.2 ± 5.0	26.5 ± 5.7	40 mg	800 mg micronized
Pabuccu et al. [20], 2022	Turkey	2021–2022	109	artificial	54	55	32.8 ± 4.2	32.3 ± 4.4	22.0 ± 2.3	22.8 ± 2.2	40 mg	180 mg vaginal gel
Ozer et al. [16], 2021	Turkey	2019	134	mNC	67	67	31.88 ± 5.20	32.4 ± 3.74	24.4 ± 4.85	23.23 ± 3.88	30 mg	8% vaginal gel
Zarei et al. [21], 2016	Iran	2014–2015	440	artificial	100	100	32.90 ± 5.10	33.51 ± 5.20	NA	NA	20 mg	800 mg vaginal suppository
Rashidi et al. [22], 2016	Iran	2015–2016	120	artificial	60	60	31.70 ± 6.48	33.27 ± 5.69	25.16 ± 2.89	24.56 ± 3.05	40 mg	800 mg vaginal suppository

BMI: Body Mass Index; SD: standard deviation; P4: progesterone; NA: not applied

## 3. Results

### 3.1. Study Selection

**Figure 1:**
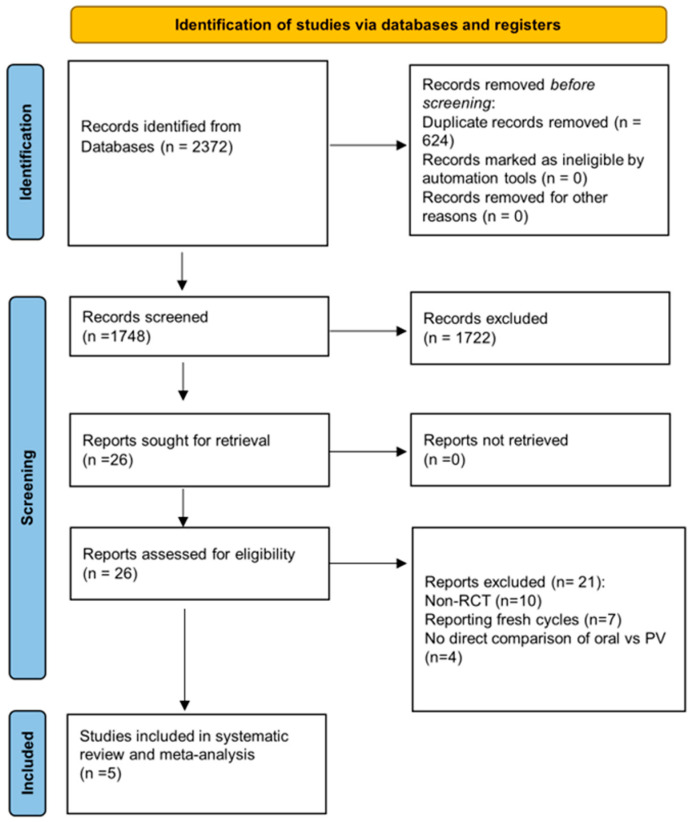
Flow chart for the systematic literature search.

The initial search retrieved 2372 studies. After duplicates were removed, 1748 studies remained for screening. Following title and abstract screening, 26 studies remained eligible for full-text screening. Finally, after full-text examination, 21 studies were excluded. In this stage, the three main reasons for exclusion were (i) non-randomized design (n = 10), (ii) reporting data on fresh cycles only (n = 7), and (iii) no direct comparison of oral versus vaginal progesterone (n = 4). References for the excluded studies are listed in Table A1, Appendix A. Therefore, five studies remained suitable for inclusion in the present systematic review and meta-analysis (Figure 1).

### 3.2. Study Characteristics

A total of 636 women who underwent a frozen–thawed embryo transfer cycle were included in the present meta-analysis (Table 1). Of the included studies, two were conducted in Turkey [16,20], two in Iran [21,22], and one in Brazil [19].

Regarding route of administration and daily dosage, three studies administered 40 mg of oral dydrogesterone [19,20,22], one study administered 30 mg of dydrogesterone [16], and another 20 mg orally [21]. As for the vaginal route, three studies administered 800 mg PV [19,20,21], one study 180 mg vaginal gel [18], and another one 8% vaginal gel [16] (Table 1).

### 3.3. Meta-Analysis Results

#### 3.3.1. Ongoing Pregnancy Rates

**Figure 2:**
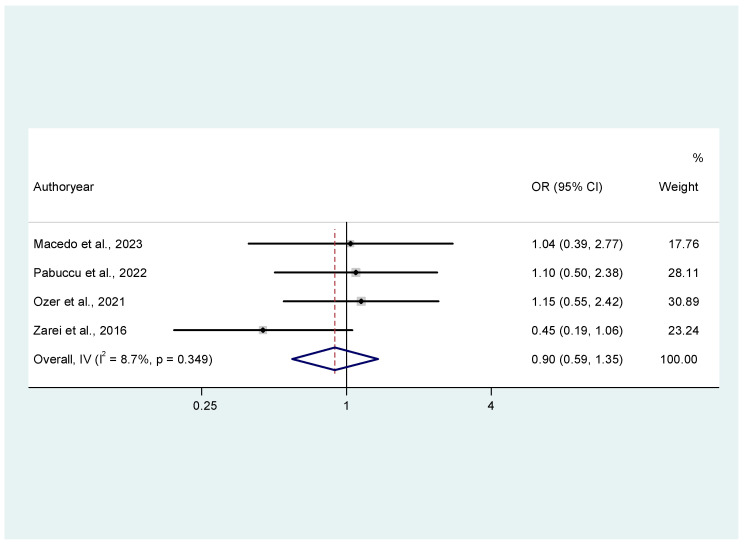
Forest plot of studies [16,19,20,21] assessing the comparison between oral dydrogesterone versus vaginal progesterone with respect to ongoing pregnancy. Results from the common effects model.

For total ongoing pregnancy events, studies did not estimate statistically significant differences between patients receiving oral dydrogesterone and patients receiving progesterone vaginally for luteal phase supplementation. Τhe pooled odds ratio (OR) was 0.90 (95% confidence interval [CI] 0.59, 1.35), indicating than women receiving oral dydrogesterone had 10% lower odds of ongoing pregnancy compared to women receiving vaginal progesterone, but the result did not reach the nominal level of statistical significance. There was low heterogeneity as indicated by I^2^ = 8.7% attributed to Zarei et al. (2016) [21] (Figure 2).

#### 3.3.2. Secondary Outcomes (Miscarriage Rates, Clinical Pregnancy Rates, Live Birth Rates)

**Figure 3:**
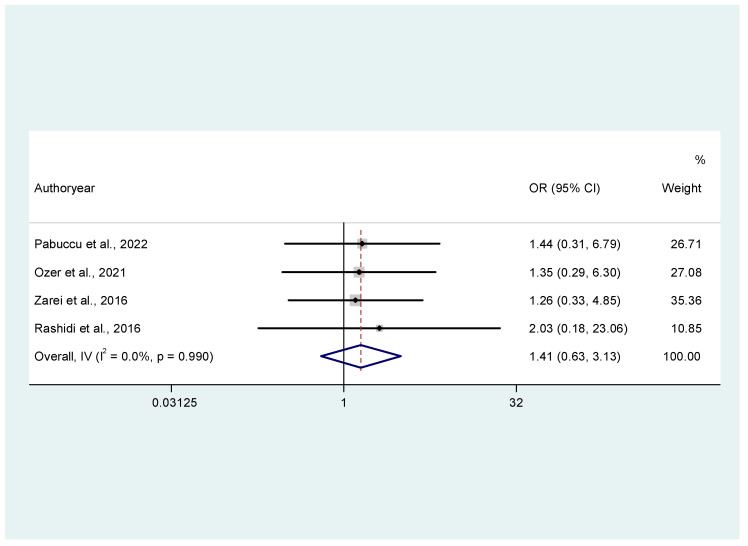
Forest plot of 4 studies [16,20,21,22] assessing the comparison between oral dydrogesterone versus vaginal progesterone and miscarriage rates. Results from common effects, inverse variance.

**Figure 4:**
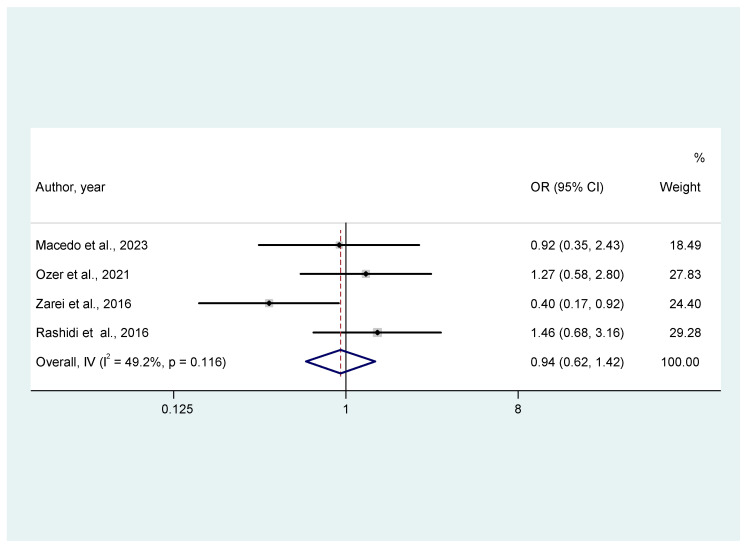
Forest plot of 4 studies [16,19,21,22] assessing the comparison between oral dydrogesterone or vaginal progesterone and clinical pregnancy rates. Results from common effects, inverse variance.

**Figure A1:**
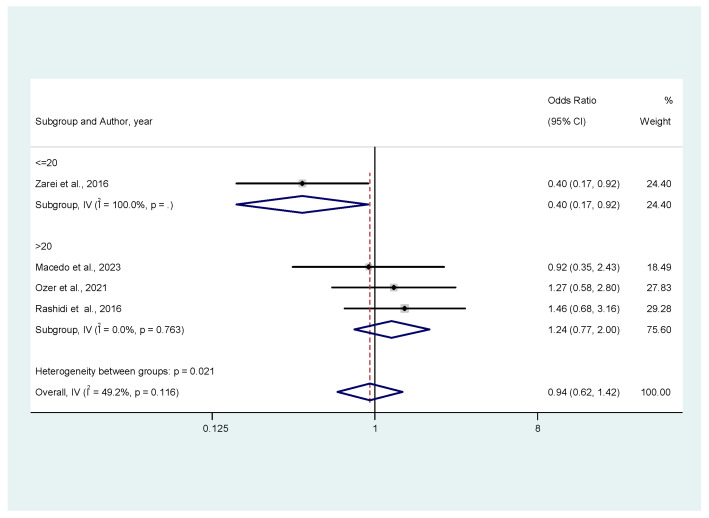
Forest plot based on sub-group analysis regarding the clinically relevant dosage of oral progesterone administration (CPR) [16,19,21,22].

**Figure A2:**
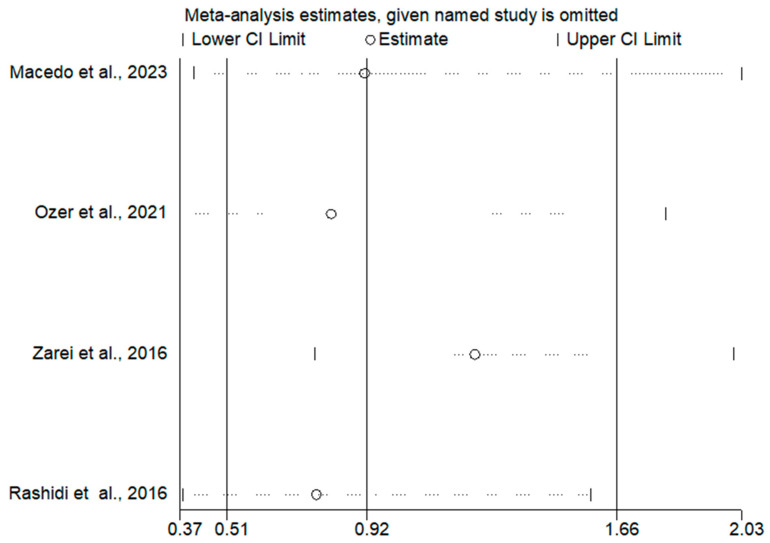
Leave-one-out forest plot of 4 studies [16,19,21,22] assessing the comparison between oral dydrogesterone or vaginal progesterone and clinical pregnancy event.

Overall, studies did not identify a statistically significant difference regarding miscarriage events between the two groups of progesterone luteal phase support. More specifically, regarding MR, oral dydrogesterone was associated with higher odds of miscarriage than vaginal progesterone, but the result was not statistically significant (OR = 1.41, 95% CI: 0.63, 3.13, I^2^ = 0, Figure 3). Moreover, regarding clinical pregnancy events, the pooled analysis resulted in an OR of 0.94 (95% CI: 0.62, 1.42), *p* = 0.11, I^2^ = 49.2%, Figure 4) between oral hydrogesterone vs. vaginal progesterone administration. The moderate heterogeneity (I^2^ = 49.2%, Figure 4) found in the analysis of clinical pregnancy events was further explored by a clinically relevant sub-grouping based on the dosage of progesterone in the experimental group. As such, two clinically relevant sub-groups were created, one with the studies where oral progesterone daily dosage was ≤ 20 mg and one with studies that have reported oral progesterone > 20 mg for luteal phase support. This sub-group was chosen since large RCTs that have established the use of oral dydrogesterone in fresh cycles have used higher dosages than 20 mg daily for the supplementation of the luteal phase [13,23]. Sub-grouping by dosage of oral progesterone administration explained the heterogeneity, as only one study has given the low dosage, and the heterogeneity in the remaining studies (group of studies with oral progesterone dosage > 20 mg daily) was 0% (Appendix A, Figure A1). Omitting this study resulted in an increase of the overall OR, suggesting favorable results for the experimental group (Appendix A, Figure A2). Sub-group analysis was conducted exclusively for the outcome of CPR, as this was the only outcome exhibiting high heterogeneity among the studies.

**Figure 5:**
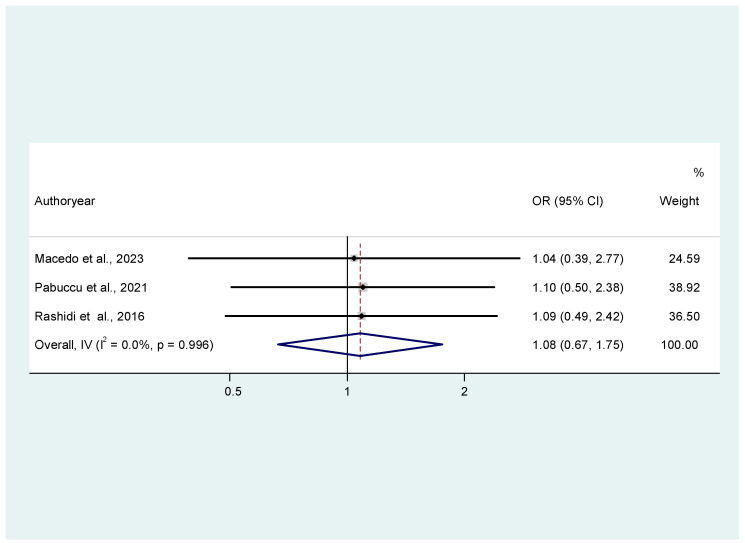
Forest plot of 3 studies [19,20,22] assessing the comparison between oral dydrogesterone or vaginal progesterone and live birth rates. Results from the common effects model, inverse variance.

Finally, regarding live birth events, patient samples regarding oral dydrogesterone in comparison to those receiving PV for luteal phase support did not statistically differ in the common effects model (OR: 1.08; (95% CI: 0.67, 1.75, *p* = 0.99, I^2^ = 0) (Figure 5).

To explore the influence of different FET protocols on the overall pooled analysis, a leave-one-out meta-analysis was performed. Excluding the study by Ozer et al. [16], the pooled OR for ongoing pregnancies decreased from 0.90 to 0.80, and for clinical pregnancies from 0.94 to 0.84, while the OR for miscarriages remained unchanged (Figure A4).

### 3.4. Risk of Bias

**Figure 6:**
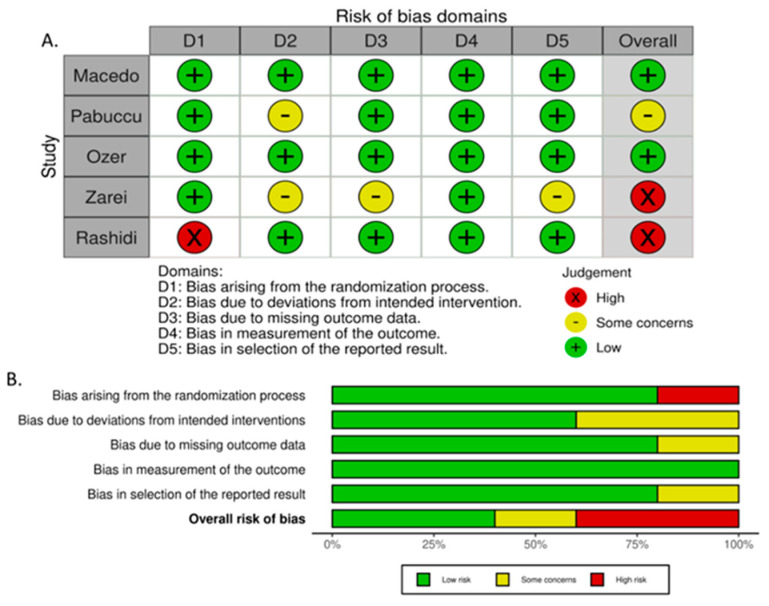
(**A**) Risk of bias per study, (**B**) risk of bias summary plot.

Two studies were considered to be of high risk of bias [21,22], two as low [16,19], and one of some concern [20] (Figure 6A,B). A comprehensive analysis of the RoB2 assessment is presented in Table A2, Appendix A.

### 3.5. Certainty of Evidence

**Figure A3:**
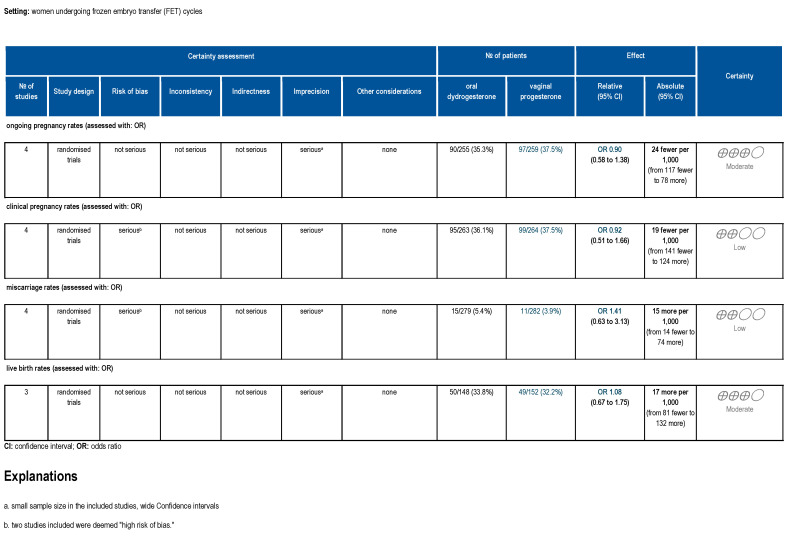
GRADE (Grading of Recommendations, Assessment, Development, and Evaluations).

**Figure A4:**
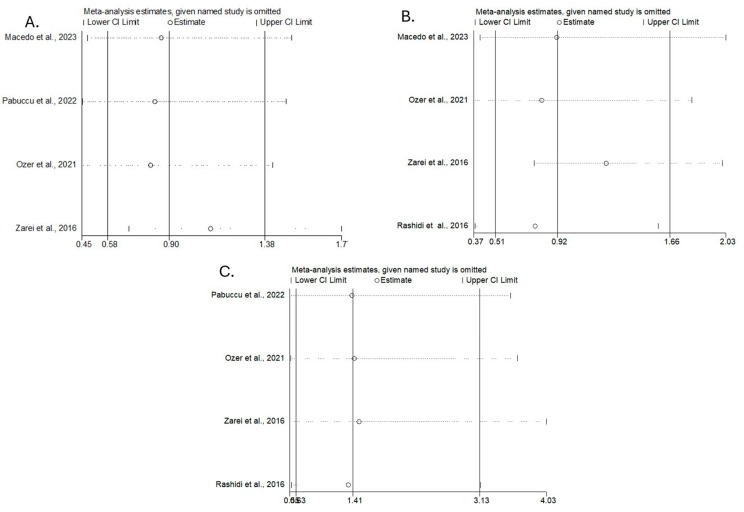
Leave-one-out forest plot to assess the influence of each study on (**A**) ongoing pregnancy rates, (**B**) clinical pregnancy rates, and (**C**) miscarriage rates [16,19,20,21,22].

Overall, the certainty of evidence using the GRADE approach was deemed moderate to low. The downgrading was mainly due to the risk of bias (two included studies were of high risk of bias), small sample sizes, the number of studies, and the wide confidence intervals reported (Figure A3, Appendix A).

## 4. Discussion

In the present systematic review and meta-analysis, we aimed to explore the efficacy of oral dydrogesterone in FET cycles compared to vaginal progesterone for luteal phase support in terms of reproductive outcomes. Overall, this pooled analysis did not yield significant differences in terms of pregnancy rates between the two groups and heterogeneity was minimal, indicating that oral dydrogesterone could be used similarly to vaginal progesterone in FET cycles. Low heterogeneity was expected, as only RCTs were included in the present analysis. Dydrogesterone is an orally given synthetic progesterone that has been used since 1960 for various menstrual disorders, for post-menopausal hormone replacement, cycle irregularity, as well as for the treatment of endometriosis [24]. Compared to natural progesterone, dydrogesterone has better oral bioavailability, with an elimination half-time of 5–7 h [25].

Several RCTs and meta-analyses have examined the use of oral dydrogesterone for luteal phase support in fresh IVF cycles. At first, oral dydrogesterone was used as an empirical supplementation for luteal phase support (LPS). The first studies that have compared dydrogesterone with vaginal progesterone have taken place in India [23,26]. In 2005, Chakravarty et al. compared the use of 20 mg dydrogesterone versus 600 mg micronized progesterone daily and concluded that similar reproductive outcomes were achieved [26]. In 2015, an updated Cochrane review, which compared the two routes of progesterone administration, reported that for CPR, the synthetic form produced better reproductive outcomes. For LBR, OPR, and MR, similar pregnancy rates were reported. Nevertheless, a substantial risk of bias and low-quality evidence was highlighted. Later, in 2020, the LOTUS II, a multi-center, open-label RCT assigned 1034 patients to receive either 30 mg dydrogesterone or 90 mg vaginal gel daily. Similar efficacy and safety were reported by the research group, suggesting that dydrogesterone may replace vaginal progesterone for LPS in the future [27]. These findings were confirmed by a later meta-analysis of nine RCTs, which included both fresh and FET IVF cycles. The researchers reported that “good quality evidence from RCTs suggests that oral dydrogesterone provided at least similar reproductive outcomes than vaginal progesterone” [28]. Nonetheless, this meta-analysis could be generalized primarily to fresh cycles, as only two RCTs regarding FET cycles were included due to the limited RCTs on FET cycles back then. As a result, data and evidence regarding FET cycles are still scarce.

Regarding adverse effects, the LOTUS trial reported a comprehensive overview of possible effects on maternal health both for dydrogesterone and vaginal progesterone. From the whole trial, 12.4% of patients in the dydrogesterone group and 16.0% of females in the micronized vaginal progesterone group suffered treatment-emerging adverse events that resulted in research termination. The liver enzyme analysis of almost every patient in both groups was normal [13]. Other research groups have outlined that vaginal progesterone could be associated with vaginal discharge and irritation [26], as well as vaginal bleeding and perineal irritation [29]. Furthermore, regarding fetal risks, malformation rates were reported to be very low when dydrogesterone was used [30]. The LOTUS I trial reported <2% congenital, familial, or genetic disorders in both the dydrogesterone and the vaginal group [13], suggesting no significant safety concerns.

Due to the increased rise of FET cycles and the consequent “freeze-all strategy” during the last decade [31], the research community has focused on the improvement of both the FET process and the freeze-all approach. Indeed, it has been reported that FET cycles are associated with a lower likelihood of OHSS occurrence and higher pregnancy rates [32]. Among the benefits of FET include the ability to preserve fertility by egg freezing in situations like cancer, and the ability to examine chromosomal abnormalities and select the best embryo for transfer through pre-implantation technology [2].

Luteal phase support is a key factor in the success of a FET cycle, and researchers have focused on finding the ideal progesterone duration, route of administration, and dosage to achieve high reproductive outcomes with minimal costs and patient satisfaction. The emphasis is currently on FET cycles in order to validate the high pregnancy rates that have been reported in fresh cycles, as dydrogesterone has been used more and more as LPS in fresh cycles in recent years. The best way to achieve this is to compare oral dydrogesterone with the gold-standard, the vaginal approach. A meta-analysis conducted by Barbosa et al. in 2018 [28] concerned mostly fresh cycles, as only two RCTs regarding FET cycles were included. Thus, the present systematic review and meta-analysis are of great significance in shedding light on the role of oral dydrogesterone in cryopreserved artificial cycles.

Our study has several limitations. First, we included a relatively small sample size, as only a small number of studies were included for each outcome. More specifically, for ongoing pregnancies, miscarriages, and clinical pregnancies, four studies were included, whereas for live births, only three studies were deemed suitable to be included in the pooled analysis. Furthermore, four of the included studies were conducted in the Middle East, and one in Brazil, which limits the generalizability of our findings to the broader infertile population worldwide. Furthermore, a sensitivity analysis or sub-group analysis according to the different forms of vaginal supplementation was not feasible, due to the small number of RCTs available. Additionally, we performed a leave-one-out meta-analysis to assess the influence of the study by Ozer et al. [16], which utilized an mNC-FET. The analysis showed slight differences in the pooled analysis. Specifically, for miscarriage rates, the OR remained unchanged. Future research based on the FET protocol regarding the use of oral dydrogesterone is needed with a larger number of included patients to better evaluate this aspect.

The main strength of our study was the inclusion of randomized trials, which, due to their inherent study design characteristics, could provide robust evidence regarding the research question. As explained above, RCTs, if successfully designed and executed, are not susceptible to confounding, which could falsely demonstrate an association between an exposure and an outcome. Additionally, the scope of our research is restricted to FET embryo transfer cycles, which have unique protocols and different endocrinological profiles than fresh cycles, such as the presence or absence of a corpus luteum. Therefore, we believe that the inclusion of only FET cycles could provide a deeper knowledge of LPS in these contexts.

Despite the inherent limitations, this study is the first meta-analysis to demonstrate the noninferiority of reproductive outcomes between oral dydrogesterone and vaginal progesterone for LPS in FET cycles. Future research should focus on direct comparisons of oral dydrogesterone with other LPS administration routes and establish standardized dosing and duration to draw more definitive conclusions.

## 5. Conclusions

Oral dydrogesterone and vaginal progesterone may be associated with similar pregnancy rates in patients undergoing FET cycles. Nevertheless, based on the few RCTs conducted so far, further research on oral dydrogesterone and FET cycles is required to assess the effectiveness, tolerability, and/or possible superiority of oral dydrogesterone over other progesterone forms for LPS. Clarifying this issue will help to establish the best possible protocol for LPS in FET cycles, in terms of effectiveness, patient safety, and satisfaction.

## Data Availability

Data are contained within the article.

## Supplementary Materials

The following supporting information can be downloaded at https://www.mdpi.com/article/10.3390/jcm14093238/s1, File S1: PRISMA 2020 Checklist.

## Abbreviations

The following abbreviations are used in this manuscript:

FET	Frozen embryo transfer
OHSS	Ovarian hyperstimulation syndrome
COS	Controlled ovarian stimulation
IM	Intramuscular
LPS	Luteal phase support
PV	Vaginal

## Appendix A

**Table A1:** Studies excluded from full-text reading (n = 21) with exclusion reasons.

Author, Year	Title	Exclusion Reasons
Vinsonneau et al., 2022	Impact of endometrial preparation on early pregnancy loss and live birth rate after frozen embryo transfer: A large multicenter cohort study (14 421 frozen cycles).	Retrospective cohort study comparing natural conception with artificial cycles, non-randomized design
Aygun et al., 2023	The Effect of Different Luteal Phase Support Applications on Clinical Pregnancy Outcomes in Frozen-Thawed Embryo Transfer.	Retrospective, non-randomized design
Liu et al., 2023	Association between duration of progesterone supplementation and clinical outcomes in artificial frozen-thawed embryo transfer cycles.	Prospective cohort study, only intramuscular progesterone is analyzed
Toriumi et al., 2023	The addition of dydrogesterone improves the outcomes of pregnant women with low progesterone levels when receiving vaginal progesterone alone as luteal support in HRT-FET cycles.	Retrospective, non-randomized design
Vidal et al., 2023	Supplementary dydrogesterone is beneficial as luteal phase support in artificial frozen-thawed embryo transfer cycles compared to micronized progesterone alone.	Retrospective, non-randomized design
Mackens et al., 2023	Individualized luteal phase support using additional oral dydrogesterone in artificially prepared frozen embryo transfer cycles: is it beneficial?	Retrospective, non-randomized design
Ikechebelu et al., 2023	A randomised control trial on oral dydrogesterone versus micronized vaginal progesterone pessary for luteal phase support in in vitro fertilization cycles.	Only fresh cycles were analyzed
Kao et al., 2022	Clinical use of aqueous subcutaneous progesterone compared with vaginal progesterone as luteal support in in vitro fertilization: A randomized controlled study in Taiwan.	Only fresh cycles were analyzed
Stadelmann et al., 2022	Vaginal progesterone as luteal phase support in natural cycle frozen-thawed embryo transfer (ProFET): protocol for a multicentre, open-label, randomised controlled trial.	Only vaginal progesterone is analyzed
Neumann et al., 2022	Dydrogesterone and 20 alpha-dihydrodydrogesterone plasma levels on day of embryo transfer and clinical outcome in an anovulatory programmed frozen-thawed embryo transfer cycle: A prospective cohort study.	Dydrogesterone levels examined, no direct comparison with vaginal progesterone
Simon et al., 2022	Comparison of two endometrial preparation methods for frozen-thawed embryo transfer in anovulatory PCOS patients: Impact on miscarriage rate.	Retrospective, non-randomized design
Ozgur et al., 2022	Dydrogesterone versus medroxyprogesterone acetate co-treatment ovarian stimulation for IVF: a matched cohort study of 236 freeze-all-IVF cycles.	Only fresh cycles were analyzed
Vuong et al., 2021	Micronized progesterone plus dydrogesterone versus micronized progesterone alone for luteal phase support in frozen-thawed cycles (MIDRONE): a prospective cohort study.	Prospective, non-randomized design
Deng et al., 2021	Efficacy of vaginal administration of Crinone versus Utrogestan combined with oral dydrogesterone tablets for luteal support in PGT freeze-thaw embryo transfer cycles. [Chinese]	Prospective, non-randomized design
Noushin et al., 2021	Dehydroepiandrosterone (DHEA) role in enhancement and maintenance of implantation (DREAM): Randomised double-blind placebo-controlled trial—Study protocol.	DHEA examined, not dydrogesterone
Devine et al., 2021	Intramuscular progesterone optimizes live birth from programmed frozen embryo transfer: a randomized clinical trial.	Intramuscular versus vaginal progesterone were compared
Atzmon et al., 2021	Comparable Outcomes Using Oral Dydrogesterone Vs. Micronized Vaginal Progesterone in Frozen Embryo Transfer: a Retrospective Cohort Study.	Retrospective, non-randomized design
Yang et al., 2020	A Phase III randomized controlled trial of oral dydrogesterone versus intravaginal progesterone gel for luteal phase support in in vitro fertilization (Lotus II): results from the Chinese mainland subpopulation.	Only fresh cycles were analyzed
Cavagna et al., 2020	Oral dydrogesterone compared to intravaginal micronized progesterone for endometrial preparation in frozen-thawed embryo transfer cycles: preliminary results of a randomized controlled trial.	Only fresh cycles were analyzed
Zargar et al., 2016	Comparison the effectiveness of oral dydrogesterone, vaginal progesterone suppository and progesterone ampule for luteal phase support on pregnancy rate during ART cycles.	Only fresh cycles were analyzed
Tomic et al., 2015	Oral dydrogesterone versus vaginal progesterone gel in the luteal phase support: Randomized controlled trial.	Only fresh cycles were analyzed

**Table A2:** Analytical risk of bias assessment.

ROB 2 DOMAINS	Macedo et al. [19]	Pabuccu et al. [20]	Ozer et al. [16]	Zarei et al. [21]	Rashidi et al. [22]
1: Bias arising from the randomization process					
**Was the allocation sequence random?**	**Yes**	**Yes**	**Yes**	**Yes**	**Yes**
Rationale/Notes (Quotes from the text to justify judgment)	The computer program used to randomize the patients into two groups was the R-Project	Therefore, 163 participants were randomly assigned based on a computer-generated list	In conclusion, a total of 134 women were assigned randomly in a ratio of 1:1 based on a computer-generated list to administer oral dydrogesterone (n = 67) or MVP (n = 67) for LPS	They were randomly assigned to four study groups using a computer-based random digit generator (each group including 100 patients)	Sequentially numbered sealed envelopes were prepared and provided by the study coordinator, according to random
Was the allocation sequence concealed until participants were enrolled and assigned to interventions?	Yes	NI	NI	NI	No
Rationale/Notes (Quotes from the text to justify judgment)	Participants received the study drugs through the institutional pharmacy, as required by the service’s protocol.	No information can be obtained from the manuscript	No information can be obtained from the manuscript	No information can be obtained from the manuscript	Envelopes were given before the intervention
Did baseline differences between intervention groups suggest a problem with the randomization process?	Probably No	No	No	No	No
Rationale/Notes (Quotes from the text to justify judgment)	Baseline differences do not suggest a problem based on Table 1	Baseline differences do not suggest a problem based on Table 1	Baseline differences do not suggest a problem based on Table 1	Baseline differences do not suggest a problem based on Table 1	Baseline differences do not suggest a problem based on Table 1
**Overall**	**Low risk**	**Low risk**	**Low risk**	**Low risk**	**High risk**
2. Bias due to deviations from intended interventions					
Were participants aware of their assigned intervention during the trial?	Yes	Yes	Yes	Yes	Yes
Rationale/Notes (Quotes from the text to justify judgment)	Open-label clinical trial. The different route of administration led to the participants knowing their group	Open-label clinical trial, technically not possible to make placebo arrangements	Open-label clinical trial	Participants were aware of their allocation due to the nature of the intervention (Oral versus vaginal)	Participants were aware of their allocation due to the nature of the intervention (Oral versus vaginal)
Were carers and people delivering the interventions aware of participants’ assigned intervention during the trial?	Yes	Yes	Yes	NI	No
Rationale/Notes (Quotes from the text to justify judgment)	Open-label clinical trial	Open-label clinical trial, technically not possible to make placebo arrangements	Open-label clinical trial	Not included throughout the text	Single-blind trial
Deviations that arose because of the trial context? (M)	No	No	No	No	No
Rationale/Notes (Quotes from the text to justify judgment)	No deviation	No deviation	No deviation	No deviation in the groups regarding the present study	No deviation
Deviations affect outcome?	Not answered because (M) is no	Not answered because (M) is no	Not answered because (M) is no	Not answered because (M) is no	Not answered because (M) is no
Rationale/Notes (Quotes from the text to justify judgment)	Not answered because (M) is no	Not answered because (M) is no	Not answered because (M) is no	Not answered because (M) is no	Not answered because (M) is no
Deviations balanced between groups?	Not answered because (M) is no	Not answered because (M) is no	Not answered because (M) is no	Not answered because (M) is no	Not answered because (M) is no
Rationale/Notes (Quotes from the text to justify judgment)	Not answered because (M) is no	Not answered because (M) is no	Not answered because (M) is no	Not answered because (M) is no	Not answered because (M) is no
Was an appropriate analysis used to estimate the effect of assignment to intervention? (S)	Probably yes	No	Yes	No	Probably yes
Rationale/Notes (Quotes from the text to justify judgment)	Based on Figure 2	All data were given as per the protocol analysis	Analyzed based on the groups to which participants were allocated	Analyzed based on the participants who adhered to the study	Based on diagram 1
If N/PN/NI to (S): Was there potential for a substantial impact (on the result) of the failure to analyze participants in the group to which they were randomized?	Not answered because (S) is PY	Probably No	Not answered because (S) is Y	Probably No	Not answered because (S) is PY
Rationale/Notes (Quotes from the text to justify judgment)	Not answered because (S) is PY	Only a small number of people were excluded from the initial allocation	Not answered because (S) is Y	We also performed multivariate logistic regression to examine the effects of the four interventions on CPR, OPR, and MR by adjustment for infertility duration, endometrial thickness, and age	Not answered because (S) is PY
**Overall**	**Low risk**	**Some concerns**	**Low risk**	**Some concerns**	**Low risk**
3. Bias due to missing outcome data					
Were data for this outcome available for all, or nearly all, participants randomized? (X)	Yes	Yes	Yes	No	Yes
Rationale/Notes (Quotes from the text to justify judgment)	Outcome data for nearly all participants and the lost to follow-up participants were lost independently of the outcome measure	No extreme losses to follow-up (93% analyzed from randomized participants)	No losses to follow-up	In the two groups concerning this meta-analysis, 10 patients were lost to follow-up, accounting for about 10%	Data were available for nearly all participants. Losses to follow-up were not important and were not associated with the outcome
If N/PN/NI to (X): Is there evidence that the result was not biased by missing outcome data? (Z)	Not answered because (X) is Yes	Not answered because (X) is Yes	Not answered because (X) is Yes	Probably no	Not answered because (X) is Yes
Rationale/Notes (Quotes from the text to justify judgment)	Not answered because (X) is Y	Not answered because (X) is Yes	Not answered because (X) is Yes	About 10% of participants in each group are lost, and no sensitivity analysis was carried out (e.g, LOCF)	Not answered because (X) is Yes
If N/PN to (Z): Could missingness in the outcome depend on its true value? (AB)	Not answered because (X) is Y	Not answered because (X) is Yes	Not answered because (X) is Yes	NI	Not answered because (X) is Yes
Rationale/Notes (Quotes from the text to justify judgment)	Not answered because (X) is Y	Not answered because (X) is Yes	Not answered because (X) is Yes	No information	Not answered because (X) is Yes
If Y/PY/NI to (AB): Is it likely that missingness in the outcome depended on its true value?	Not answered because (X) is Y	Not answered because (X) is Yes	Not answered because (X) is Yes	NI	Not answered because (X) is Yes
Rationale/Notes (Quotes from the text to justify judgment)	Not answered because (X) is Y	Not answered because (X) is Yes	Not answered because (X) is Yes	No information	Not answered because (X) is Yes
**Overall**	**Low risk**	**Low risk**	**Low risk**	**Some concerns**	**Low risk**
4. Bias in measurement of the outcome					
Was the method of measuring the outcome inappropriate? (AG)	No	No	No	No	No
Rationale/Notes (Quotes from the text to justify judgment)	Clinical pregnancy, ongoing pregnancy, and miscarriages are objectively measured through ultrasound. Live births are objectively measured	Clinical pregnancy, ongoing pregnancy, and miscarriages are objectively measured through ultrasound. Live births are objectively measured	Clinical pregnancy, ongoing pregnancy, and miscarriages are objectively measured through ultrasound. Live births are objectively measured	Clinical pregnancy, ongoing pregnancy, and miscarriages are objectively measured through ultrasound. Live births are objectively measured	Clinical pregnancy, ongoing pregnancy, and miscarriages are objectively measured through ultrasound. Live births are objectively measured
Could measurement or ascertainment of the outcome have differed between intervention groups? (AI)	No	No	No	No	No
Rationale/Notes (Quotes from the text to justify judgment)	Clinical pregnancy, ongoing pregnancy, and miscarriages are objectively measured through ultrasound. Live births are objectively measured	Clinical pregnancy, ongoing pregnancy, and miscarriages are objectively measured through ultrasound. Live births are objectively measured	Clinical pregnancy, ongoing pregnancy, and miscarriages are objectively measured through ultrasound. Live births are objectively measured	Clinical pregnancy, ongoing pregnancy, and miscarriages are objectively measured through ultrasound. Live births are objectively measured	Clinical pregnancy, ongoing pregnancy, and miscarriages are objectively measured through ultrasound. Live births are objectively measured
If N/PN/NI to (AG, AI): Were outcome assessors aware of the intervention received by study participants? (AK)	Yes	Yes	Yes	Yes	No
Rationale/Notes (Quotes from the text to justify judgment)	Open-label	Open-label	Open-label	Open-label	Trial blinded to assessors of the outcome
If Y/PY/NI to (AK): Could assessment of the outcome have been influenced by knowledge of intervention received? (AM)	No	No	No	No	Not answered because (AK) is No
Rationale/Notes (Quotes from the text to justify judgment)	Outcomes objectively measured	Outcomes objectively measured	Outcomes objectively measured	Outcomes objectively measured	Not answered because (AK) is No
If Y/PY/NI to (AM): Is it likely that assessment of the outcome was influenced by knowledge of intervention received?	Not answered because (AM) are No	Not answered because (AM) are No	Not answered because (AM) are No	Not answered because (AM) are No	Not answered because (AK) is No
Rationale/Notes (Quotes from the text to justify judgment)	Not answered because (AM) are No	Not answered because (AM) are No	Not answered because (AM) are No	Not answered because (AM) are No	Not answered because (AK) is No
**Overall**	**Low risk**	**Low risk**	**Low risk**	**Low risk**	**Low risk**
5. Bias in selection of the reported result					
Were the data that produced this result analyzed in accordance with a pre-specified analysis plan that was finalized before unblinded outcome data were available for analysis?	Probably Yes	Probably Yes	0	NI	Probably Yes
Rationale/Notes (Quotes from the text to justify judgment)	Document Number: 3.453.065, Rapporteurship on 13 July 2019, and Brazilian Registry of Clinical Trials (ReBEC). UTN (Universal Trial Number): U1111-1247-1845, date: 31 March 2020	The study was registered at clinicaltrials.gov (NCT03948022)	The study is also registered at ClinicalTrials.gov (NCT04124913)	No registration number	The registration number of the trial was IRCT201406255181N15.
Is the numerical result being assessed likely to have been selected?	Probably No	Probably No	Probably No	Probably No	Probably No
Rationale/Notes (Quotes from the text to justify judgment)	Reasonable outcomes are presented	Reasonable outcomes are presented	Reasonable outcomes are presented	Reasonable outcomes are presented	Reasonable outcomes are presented
**Overall**	**Low risk**	**Low risk**	**Low risk**	**Some concerns**	**Low risk**
OVERALL ASSESSMENT	**Low risk**	**Some concerns**	**Low risk**	**High risk**	**High risk**
